# Patterns of Pretreatment Diagnostic Assessment in Patients Treated with Stereotactic Body Radiation Therapy (SBRT) for Non-Small Cell Lung Cancer (NSCLC): Special Characteristics in the COVID Pandemic and Influence on Outcomes

**DOI:** 10.3390/curroncol29020092

**Published:** 2022-02-13

**Authors:** Felix-Nikolai Oschinka Jegor Habermann, Daniela Schmitt, Thomas Failing, Jann Fischer, David Alexander Ziegler, Laura Anna Fischer, Niklas Josua Alt, Julian Muster, Sandra Donath, Andrea Hille, Markus Anton Schirmer, Manuel Guhlich, Rami A. El Shafie, Stefan Rieken, Martin Leu, Leif Hendrik Dröge

**Affiliations:** 1 Department of Radiotherapy and Radiation Oncology, University Medical Center Göttingen, Robert-Koch-Str. 40, 37075 Göttingen, Germany; felix.habermann@med.uni-goettingen.de (F.-N.O.J.H.); daniela.schmitt@med.uni-goettingen.de (D.S.); thomas.failing@med.uni-goettingen.de (T.F.); jann.fischer@med.uni-goettingen.de (J.F.); alexander.ziegler@med.uni-goettingen.de (D.A.Z.); laura-anna.fischer@med.uni-goettingen.de (L.A.F.); niklasjosua.alt@stud.uni-goettingen.de (N.J.A.); julian.muster@stud.uni-goettingen.de (J.M.); sandra.donath@med.uni-goettingen.de (S.D.); ahille@med.uni-goettingen.de (A.H.); mschirmer@med.uni-goettingen.de (M.A.S.); manuel.guhlich@med.uni-goettingen.de (M.G.); rami.elshafie@med.uni-goettingen.de (R.A.E.S.); stefan.rieken@med.uni-goettingen.de (S.R.); martin.leu@med.uni-goettingen.de (M.L.); 2 Comprehensive Cancer Center Niedersachsen (CCC-N), University Medical Center Göttingen, Robert-Koch-Str. 40, 37075 Göttingen, Germany

**Keywords:** non-small cell lung cancer, stereotactic body radiation therapy, diagnostic assessment, staging examinations, positron emission tomography/computed tomography scan, pandemic, coronavirus disease 2019, outcomes

## Abstract

The pandemic raised a discussion about the postponement of medical interventions for non-small cell lung cancer (NSCLC). We analyzed the characteristics of pretreatment diagnostic assessment in the pandemic and the influence of diagnostic assessment on outcomes. A total of 96 patients with stereotactic body radiation therapy (SBRT) for NSCLC were included. The number of patients increased from mean 0.9 (2012–2019) to 1.45 per month in the COVID era (*p* < 0.05). Pandemic-related factors (contact reduction, limited intensive care unit resources) might have influenced clinical decision making towards SBRT. The time from pretreatment assessment (multidisciplinary tumor board decision, bronchoscopy, planning CT) to SBRT was longer during the COVID period (*p* < 0.05). Reduced services, staff shortage, or appointment management to mitigate infection risks might explain this finding. Overall survival, progression-free survival, locoregional progression-free survival, and distant progression-free survival were superior in patients who received a PET/CT scan prior to SBRT (*p* < 0.05). This supports that SBRT guidelines advocate the acquisition of a PET/CT scan. A longer time from PET/CT scan/conventional staging to SBRT (<10 vs. ≥10 weeks) was associated with worse locoregional control (*p* < 0.05). The postponement of diagnostic or therapeutic measures in the pandemic should be discussed cautiously. Patient- and tumor-related features should be evaluated in detail.

## 1. Introduction

Lung cancer is the most frequent cause of cancer death worldwide [1]. In 2020, the disease resulted in 1.8 million deaths worldwide [1]. About 15–25% of the patients are diagnosed with localized stages I-II [2] (p. 62), and [3,4]. A relevant proportion of the non-small cell lung cancer (NSCLC) patients cannot undergo surgical resection, either due to comorbidities or due to patient refusal [5]. In these patients, stereotactic body radiation therapy (SBRT) represents a therapeutic option [5,6].

Before SBRT, an adequate patient selection is crucial to ensure optimal treatment delivery and outcomes [7]. Previous studies indicated that pretreatment assessment (including the latency from diagnostic procedures to the initiation of treatment) even has a relevant influence on outcomes [8,9]. In the current guidelines, the pretreatment diagnostic procedures and treatment delivery are discussed in detail [10,11,12,13]. These aspects include the indications for less-invasive (e.g., cranial magnetic resonance imaging (cMRI) scan or positron emission tomography/computed tomography (PET/CT) scan) and more-invasive staging procedures (e.g., endoscopic examination) [10,12,13]. However, Cornwell et al. point out that there is limited evidence to define clear standards [11].

At the same time, the coronavirus pandemic (COVID) situation in 2020 and 2021 has set the focus on the management of medical resources and on the efforts to reduce contacts to slow the viral spread [14]. A discussion was triggered on the management of cancer and on the risks and priorities of medical procedures [14,15,16]. However, Guckenberger et al. emphasized that there is very limited evidence to guide the recommendations in the context of the pandemic [15]. It can be hypothesized that the pandemic situation affects the patterns of pretreatment diagnostic assessment and, thus, could have a relevant impact on oncological treatment strategies and patient outcomes.

In this study, we integrate both of the aforementioned aspects. First, we compared the pretreatment assessment in patients who received SBRT for NSCLC before the pandemic and in patients who were treated during the pandemic. Second, we analyzed the influence of pretreatment diagnostic assessment on outcomes in all the patients who were treated at our Lung Cancer Centre (both during the pre-COVID era and COVID era).

## 2. Patients and Methods

### 2.1. Patients and Study Design

**Figure 1:**
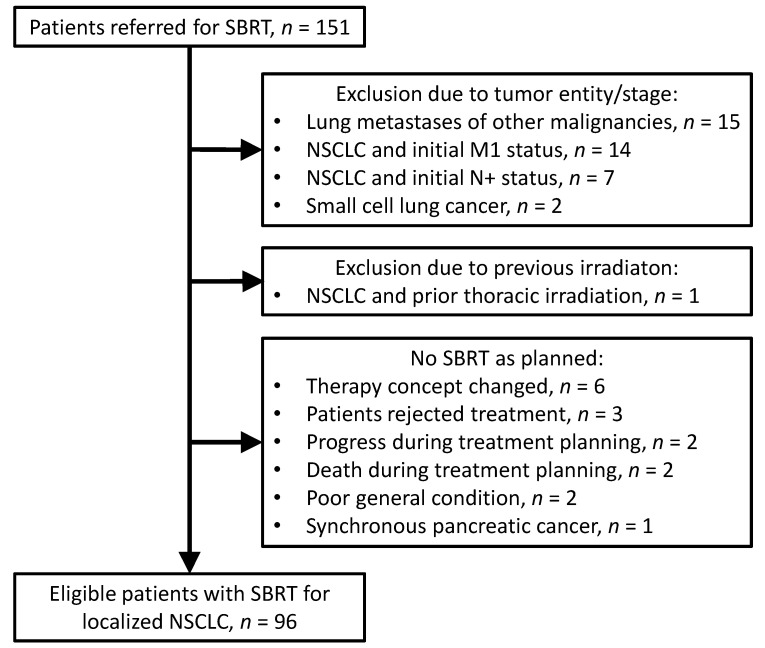
Flow chart. The chart informs about the selection of the 96 patients for outcome analysis. SBRT: stereotactic body radiation therapy; NSCLC: non-small cell lung cancer.

We identified patients who were referred for SBRT to our institution. In total, 151 patients were assessed for eligibility. Among these, 96 patients with SBRT for localized NSCLC (start of SBRT, December 2012 to January 2021) were included in the study for further analysis. A total of 39 patients were excluded due to tumor entity, tumor stage, or previous irradiation. In 16 patients, SBRT was not applied as planned. Please see Figure 1 for further details. The staging procedures and treatment procedures were conducted in accordance with the national and international guidelines [10,12,13,17]. The treatment decisions were based on the multidisciplinary tumor board of the regional Lung Cancer Centre. The study was conducted after ethical approval by the ethics committee of the University Medical Center Göttingen (number 3/10/20).

### 2.2. Stereotactic Body Radiation Therapy

Patients received four-dimensional computed tomography (CT). A respiration belt was used to detect the breathing excursions. Patients were immobilized in supine position with customized positioning devices. In 28 patients, the MacroMedics Omniboard^TM^ system was applied. We used the CT scanners Philips Gemini TF TOF 16 (*n* = 24 patients), Philips Ingenuity Flex (*n* = 3 patients), and Philips Brilliance Big Bore (*n* = 69 patients). The scans were acquired with a slice thickness of 2 mm (*n* = 4 patients), 3 mm (*n* = 90 patients), or 5 mm (*n* = 2 patients). The gross tumor volume was contoured in each of the respiratory phases. The internal target volume was generated by combining the gross tumor volumes of the respiratory phases. The margins for the planning target volume were set on an individual basis by the treating physician (range, 0.3 cm–1.0 cm). Treatment plans were generated using the treatment planning system Eclipse (Varian Medical Systems, Palo Alto, CA, USA). The Eclipse^TM^ versions 10.0 (from December 2012), 11.1 (from October 2013), 13.5 (from October 2014), and 15.6 (from June 2020) were used. The dose calculation algorithms were Acuros (*n* = 86 patients) and AAA (*n* = 10 patients). The dose was prescribed to the 80% isodose (*n* = 78 patients) or to the 95% isodose (*n* = 18 patients). SBRT was delivered with a linear accelerator (Varian Clinac 2300 CD) and daily cone beam CT imaging.

### 2.3. Statistical Methods

First, the characteristics of pretreatment assessment (patients with SBRT in the pre-COVID era vs. patients in the COVID era) were compared. Second, we used univariable Cox regression analysis to test for an influence of pretreatment diagnostic assessment on outcomes. The survival times were calculated from the day of the start of SBRT. We analyzed overall survival (OS; event: patient death due to any cause), progression-free survival (PFS, events: patient death, tumor progression), locoregional progression-free survival (LRPFS, events: patient death, locoregional progression), locoregional control (LRC, event: locoregional progression), distant progression-free survival (DPFS, events: patient death, distant progression), and distant control (DC, event: distant metastases). Tumor progression was evaluated in the context of the multidisciplinary tumor board of the regional Lung Cancer Centre. The evaluation was based on the RECIST criteria [18]. The software SPSS version 27 (IBM Corp., Armonk, NY, USA) was used for the Cox regression analysis and for the comparison of patient groups (Pearson’s chi-squared test and Mann-Whitney U test). The software R version 4.1.0 with the plugin KMWin version 1.53 was used to draw the survival curves, including the log-rank test [19]. In this exploratory analysis, *p*-values < 0.05 were considered statistically significant. Additionally, the *p*-values of the Cox regression analysis were adjusted using the Benjamini Hochberg procedure. We assigned ranks to the *p*-values in ascending order starting from the lowest. We chose a false discovery rate of 0.2. We identified the largest raw *p*-value which was smaller than the corresponding critical value for the same rank. All the *p*-values with smaller ranks were considered statistically significant [20].

## 3. Results

### 3.1. Baseline Characteristics

The median patient age was 73.0 years (range 57.2–89.8). The study cohort consisted of 32 female patients (33.3%) and 64 male patients (66.7%). The predominant dose fractionation schedules were 60 Gy in 8 fractions of 7.5 Gy (*n* = 46 patients, 47.9%) and 55 Gy in 5 fractions of 11.0 Gy (*n* = 37 patients, 38.5%). The predominant radiotherapy technique was volumetric modulated arc therapy (*n* = 83 patients, 86.5%). Please see Table 1 and Table S1 for further details.

### 3.2. Comparison of Pretreatment Assessment in the pre-COVID Era and in the COVID Era

The characteristics of the pretreatment assessment were compared between patients in the pre-COVID era (start of SBRT in 2012–2019, *n* = 77) and patients in the COVID era (start of SBRT in 2020–2021, *n* = 19). The number of patients with SBRT per month was significantly higher in the COVID era (1.5 vs. 0.9 patients per month, *p* = 0.04). Moreover, the time from pretreatment assessment to the first day of SBRT was longer in patients who were treated in the COVID era. We found significant differences for the parameters multidisciplinary tumor board decision to SBRT (*p* = 0.005), bronchoscopy to SBRT (*p* = 0.04), and planning CT to SBRT (*p* < 0.001). For the parameters with significant differences, we additionally compared patients treated from 2018–2019 vs. patients treated from 2020–2021 to evaluate the influence of the pandemic on more recent patient management. Here, the number of patients per month was higher and the median times from prediagnostic assessment to SBRT were longer in the COVID era. However, there was only a statistically significant difference for the time from planning CT to SBRT (*p* < 0.001). Please see Table 2 and Table S2 for further details.

### 3.3. Influence of Pretreatment Diagnostic Assessment on Outcomes

**Figure 2:**
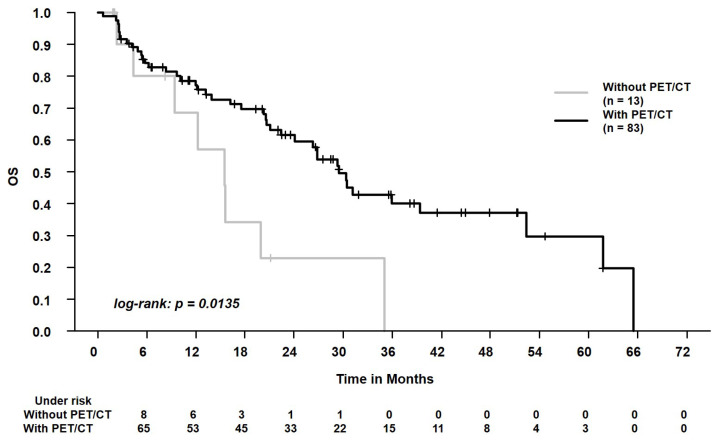
Overall survival (OS) in patients who were staged with positron emission tomography/computed tomography (with PET) vs. patients who were staged with conventional chest CT scan (without PET). The 2-year OS was 61.5% vs. 22.9%.

**Figure 3:**
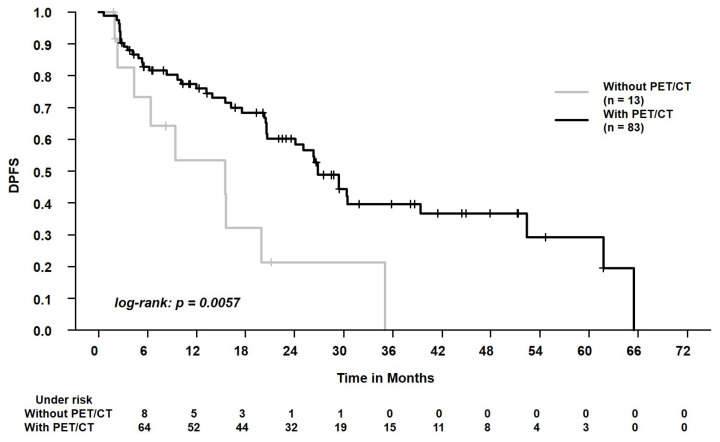
Distant progression-free survival (DPFS) in patients who were staged with positron emission tomography/computed tomography (with PET) vs. patients who were staged with conventional chest CT scan (without PET). The 2-year DPFS was 60.3% vs. 21.4%.

**Figure 4:**
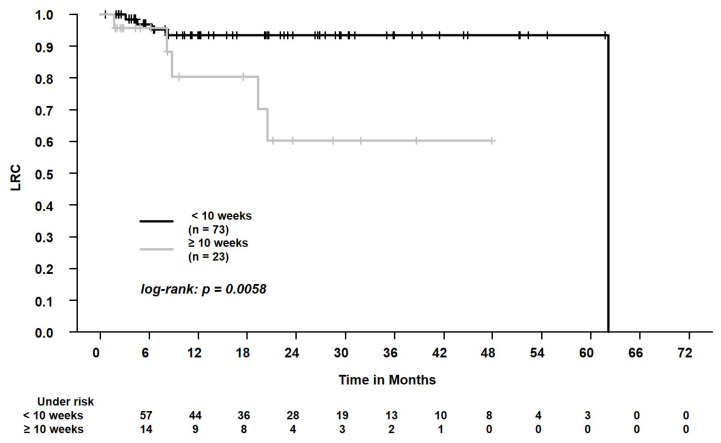
Locoregional control (LRC) in patients who were staged with positron emission tomography/computed tomography or chest CT scan < 10 weeks vs. ≥ 10 weeks before the start of SBRT. The 2-year LRC was 94.5% vs. 58.4%.

**Figure 5:**
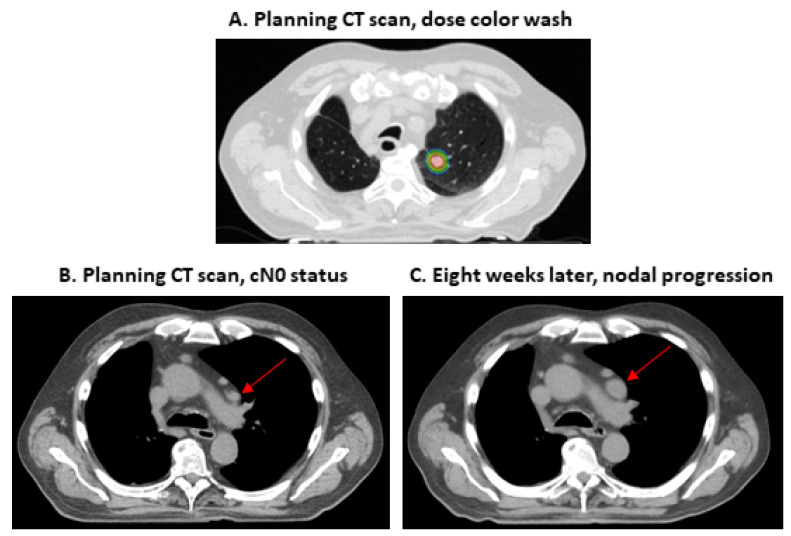
Clinical example of a patient without positron emission tomography/computed tomography staging and subsequent rapid regional progression. Male patient diagnosed with stage IA2 non-small cell lung cancer of the left upper lobe. The patient was staged with conventional imaging (computed tomography scan of the upper and lower body, cranial magnetic resonance imaging and bronchoscopy, image (**B**) shows the initial status). The patient was treated with 55 Gy in 5 fractions (prescribed to the 80% isodose; image (**A**) shows the dose from 55 Gy [blue] to 68.75 Gy [red]). A CT-scan 2 months later (image (**C**)) showed good response of the primary tumor, but revealed the progression of a mediastinal lymph node (growth from 1.5 cm × 0.9 cm to 2.3 × 1.5 cm, red arrows in images (**B**,**C**)).

In the whole study cohort, the median follow-up was 18.4 months (range, 0.6–65.5 months). The 2-year overall survival and progression-free survival were 56.8% and 53.7%, respectively. During follow-up, 50/96 patients (52.1%) died. Local and regional progression occurred in 6/96 patients (6.3%) and 7/96 patients (7.3%), respectively. During follow-up, distant metastases were registered in 11/96 patients (11.5%). We found that patients with PET/CT for tumor staging experienced better OS, PFS, LRPFS, and DPFS (each, *p* < 0.05, Table 3). In the whole cohort, patients who were staged with PET/CT or chest CT scan ≥ 10 weeks before SBRT experienced worse LRC than patients who were staged with PET/CT or chest CT scan < 10 weeks before SBRT (*p* = 0.01, Table 3). When analyzing only patients who received a PET/CT scan (*n* = 83), the time to SBRT (≥10 weeks vs. <10 weeks) affected LRC, too (*p* = 0.01, Table 3). Please see Figure 2, Figure 3 and Figure 4 for the Kaplan–Meier plots and the log-rank tests. Please see Figure 5 for a clinical example of a patient who was staged without PET/CT and presented with rapid tumor progression after SBRT. Please see Table S3 for the adjusted *p* values of the Cox regression analysis using the Benjamini Hochberg procedure. Here, the statistical significance was retained.

## 4. Discussion

Lung cancer is the most frequent cause of cancer death worldwide [1]. About 15–25% of the patients are diagnosed with localized stages I-II [2] (p. 62) and [3,4]. SBRT is a therapeutic option in patients who are not candidates for oncological tumor resection [5]. In the current guidelines, the indications for less-invasive (e.g., cMRI scan and PET/CT scan) and more-invasive staging procedures (e.g., endoscopic examination) are discussed in detail, though there is limited scientific evidence to establish clear standards [10,11,12,13]. The indication for brain imaging can serve as an example. Here, Videtic et al. and Schneider et al. recommend brain imaging for patients who are suspected to have multiple primary lung cancers [10,12]. Guckenberger et al. advise against brain imaging in patients with early-stage cN0 tumors [13]. These recommendations leave room for individual clinical decision making. At the same time, the COVID pandemic has brought into focus the management of medical resources and efforts to reduce contacts to slow the viral spread [14]. Here, Guckenberger et al. emphasized that there is very limited evidence on the risks and priorities of medical interventions in the context of radiation oncology [15]. In this study, we compared the pretreatment assessment in patients with SBRT for NSCLC before the pandemic and during the pandemic. Moreover, we analyzed the influence of pretreatment diagnostic assessment on outcomes.

First, we found that the number of patients who received SBRT for NSCLC at our Lung Cancer Centre increased from a mean of 0.9 to 1.45 per month in the COVID era. During the pandemic, the contact reduction and the allocation of intensive care unit resources are of major importance [23]. Here, SBRT has advantages when compared to surgery [24]. Additionally, SBRT is favorable during the pandemic because it is applied in a few fractions and can be delivered as a convenient outpatient procedure [25]. Couñago et al. emphasized that SBRT is an important option during the pandemic, both for operable and inoperable NSCLC patients [15,26]. These advantages might have influenced clinical decision making towards SBRT during the COVID period. This could explain the increasing number of SBRT patients. However, due to the retrospective nature of the study, further influencing factors cannot be excluded (e.g., the availability of SBRT in the context of the Lung Cancer Centre). Additionally, when comparing recent patients (SBRT in 2018–2019) with patients from the COVID era, some of the observed effects were not statistically significant. Thus, the findings should be interpreted cautiously.

Furthermore, we found that the time from pretreatment assessment to the initiation of SBRT was longer during the COVID period. When comparing the pre-COVID era and the COVID era, the time was 2.4 weeks (multidisciplinary tumor board decision), 1.4 weeks (bronchoscopy), and 1.3 weeks (planning CT) longer. Combs et al. stated that the COVID pandemic requires efforts to set priorities for the available resources [14]. Jazieh et al. reported the results from a global survey on cancer care during the pandemic [27]. The authors reported that 55% of the treatment units reduced services and that 18% of the centers suffered from staff shortage [27]. Even if technical and personnel resources are sufficient, an appointment management might minimize the risks of infections via the reduction of patient travel or via the reduction of contacts with COVID-positive patients [15,28,29]. Altogether, these aspects might explain the longer times from pretreatment assessment to SBRT in the presented study. However, for the period of the present study, there were no systematic data available on technical (e.g., downtimes of machines) or personnel resources (e.g., absences from work due to illness). It can be stated that, during the pandemic, a relevant number of hospital beds were used for COVID patients. According to local practice, bronchoscopies and SBRT were regularly conducted under stationary conditions. The shortage of hospital beds could explain the longer time from bronchoscopies and planning CT scan to SBRT. Moreover, the number of attending physicians was reduced in the multidisciplinary tumor boards to reduce the risks of COVID infections. This could have resulted in slower information transfer with a delay in subsequent treatments. This could explain the longer time from tumor board decisions to the start of SBRT in the COVID era. In summary, further studies would be necessary to clarify the reasons for the differences between the pre-COVID era and the COVID era.

The question arises whether a reduced use of resources negatively affects outcomes in SBRT patients. We found that patients with PET/CT scan prior to SBRT experienced better OS, PFS, LRPFS, and DPFS. There was a strong effect of this parameter with hazard ratios from 0.34 to 0.38. Interestingly, as Guckenberger et al. stated in 2017, there is no sufficient scientific evidence to set a clear standard on PET/CT acquisition before SBRT [13]. Videtic et al. recommended a PET/CT scan in cases of multiple primary lung cancers, while Schneider et al. recommend the diagnostic scan in patients with centrally located tumors, larger tumors, and in the absence of histological tumor confirmation [10,12]. At the same time, previous studies demonstrated that a PET/CT scan, when compared to conventional approaches, detects unexpected metastases in around 10% of the patients [8,30]. Moreover, Wang et al. reported a negative predictive value for mediastinal lymph node involvement of 0.93 [31]. Van Tinteren et al. conducted a randomized trial on patients with potentially resectable localized NSCLC [8]. When integrating a PET/CT scan as staging examination, they even found that 20% of the candidates were not suitable for surgery, e.g., due to extensive mediastinal disease or due to distant metastases [8]. Our study supports that these findings can be extrapolated to patients who are referred to SBRT [32]. The PET/CT scans have a crucial role in identifying patients who may profit from local treatment [32]. The clinical example (Figure 5) of a patient who received conventional staging and experienced nodal progression after a very short period of 2 months illustrates the relevance for this patient population. This supports that SBRT guidelines advocate the acquisition of a PET/CT scan prior to SBRT due to the prognostic relevance and to minimize the risk of under-treating patients [10,12,13]. However, due to the retrospective study design, the acquisition of PET/CT scans vs. conventional staging was conducted non-randomized. Thus, additional parameters might have distorted our study’s results.

Lastly, we analyzed whether longer times from pretreatment assessment to SBRT negatively affect outcomes. Here, we found worse LRC for patients with conventional staging and PET/CT scans ≥ 10 weeks before SBRT. In 2017, Guckenberger et al. designed a questionnaire and reported the answers of numerous experts for SBRT [13]. The acceptable interval from PET/CT scan to SBRT was in the range of 1 to 6 months [13]. Previous studies on patients with NSCLC (>50% of patients in stage III) demonstrated that tumor progression occurs when treatment is delayed for several weeks [9]. In 2020, Guckenberger et al. developed practice recommendations for lung cancer radiotherapy during the COVID pandemic based on expert options [15]. For stage I NSCLC, there was no consensus whether SBRT initiation can safely be postponed [15]. The experts recommended that further parameters (e.g., tumor growth rate, patient preference, T1 vs. T2 stage) should be integrated in the decision process [15]. In the general population of patients with SBRT at our Lung Cancer Centre, we found worse LRC when staging was acquired ≥10 weeks before SBRT. Interestingly, Everitt et al. reported a comparable doubling time of about 10 weeks (66 days) for fluorodeoxyglucose-avid NSCLC [33]. This indicates that 10 weeks from PET/CT scan or diagnostic CT scan to SBRT could be a pragmatic cut-off when deciding whether staging examinations should be repeated prior to SBRT initiation [22]. The PACIFIC-4 trial on patients with lung SBRT is currently recruiting and applies this cut-off as an inclusion criterion [22]. However, these aspects should be interpreted cautiously. We analyzed a relatively small patient cohort and the results may be prone to biases when choosing the pragmatic cut-off of 10 weeks. Additionally, since the time from histopathological diagnosis or from staging examinations to SBRT may have an influence on patient outcomes, the decision to calculate the survival times starting from the first day of SBRT is debatable. Moreover, we chose a significance level of 0.05. Though statistical significance was retained when adjusting for multiple comparisons, the results should only be understood as hypothesis generating. Further studies with higher patient numbers should be conducted to draw firm conclusions. Nevertheless, in the light of possible tumor progression and worse outcomes, the postponement of diagnostic or therapeutic procedures in the context of the COVID pandemic should be discussed cautiously [15]. Patient- and tumor-related features should be evaluated in detail before postponement [34].

## 5. Conclusions

A total of 96 patients with SBRT for NSCLC were retrospectively analyzed. We compared characteristics in the pre-COVID era (2012–2019) and characteristics in the COVID era (2020–2021). First, we found that the number of patients who received SBRT at our Lung Cancer Centre increased from a mean of 0.9 to 1.45 per month in the COVID era. Here, the consideration of pandemic-related factors (contact reduction, intensive care unit resources) might have influenced clinical decision making towards SBRT [26]. Second, the time from pretreatment assessment (multidisciplinary tumor board decision, bronchoscopy, planning CT) to the initiation of SBRT was longer during the COVID period. This might be explained by reduced services, staff shortage, or appointment management to mitigate infection risks [27]. In the whole cohort, the oncological outcomes were better in patients who received a PET/CT scan prior to SBRT. This supports that SBRT guidelines advocate the acquisition of a PET/CT scan prior to SBRT due to the prognostic relevance and to minimize the risk of under-treating patients [10,12,13]. A longer time from PET/CT scan or conventional staging to the initiation of SBRT (<10 vs. ≥10 weeks) was associated with worse outcomes. Thus, the postponement of diagnostic or therapeutic procedures in the context of the COVID pandemic should be discussed cautiously [15]. Patient- and tumor-related features should be evaluated in detail [34].

## Figures and Tables

**Table 1:** Patient baseline characteristics. If not otherwise specified, patient numbers are given with percentage values in brackets. ECOG: eastern cooperative oncology performance status. SBRT: stereotactic body radiation therapy. 3DCRT: 3D conformal radiotherapy. IMRT: intensity-modulated radiotherapy. VMAT: volumetric modulated arc therapy. ^1^ TNM, 8th Edition. ^2^ One patient presented with two separate tumors (left lung, cT1a squamous cell cancer; right lung, cT1b tumor, without histological confirmation). After multidisciplinary tumor board evaluation, the tumors were considered as two synchronous primary malignancies. SBRT was applied to both tumors (left lung, 44 Gy in 8 fractions; right lung, 55 Gy in 5 fractions). The patient is counted only once in Table 1, as denoted. ^3^ Four patients received 54 Gy in 18 fractions. Recent consensus reports define SBRT as a treatment with a maximum number of 12 fractions [21]. Since this study mainly focused on pre-treatment diagnostic assessment, not on the technical aspects of SBRT, we decided to include these four patients. ^4^ We analyzed whether there was a difference in the proportion of patients with T4 tumors during the pre-COVID era (*n* = 3/77) and the COVID era (*n* = 3/19). There was no statistical difference between groups (*p* = 0.055).

Parameter	
Age, years, median (min–max)	73.0 (57.2–89.8)
ECOG, median (min–max)	1 (0–4)
Gender	
Female	32 (33.3)
Male	64 (66.7)
Histology	
Adenocarcinoma	37 (38.5)
Squamous cell cancer	36 (37.5) ^2^
Large cell carcinoma	1 (1.0)
Carcinoma, not otherwise specified	4 (4.2)
Without histological confirmation	18 (18.8)
Tumor stage ^1^	
IA	58 (60.4) ^2^
IB	17 (17.7)
IIA	5 (5.2)
IIB	10 (10.4)
IIIA	6 (6.3)
cT category ^1,4^	
cT1	58 (60.4) ^2^
cT2	22 (22.9)
cT3	10 (10.4)
cT4	6 (6.3)
SBRT, total dose [Gy]/number of fractions	
60/8	46 (47.9)
55/5	37 (38.5) ^2^
54/3	6 (6.3)
54/18 ^3^	4 (4.2)
50/10	2 (2.1)
60/12	1 (1.0)
SBRT, technique	
3DCRT	10 (10.4)
IMRT	3 (3.1)
VMAT	83 (86.5) ^2^

**Table 2:** Comparison of pretreatment assessment between patients treated in the pre-COVID (Coronavirus Disease) era (here defined from 2012–2019) and in the COVID era. The times for the parameters were calculated to the first day of SBRT (stereotactic body radiation therapy). PET/CT: positron emission tomography/computed tomography. CT: computed tomography. cMRI: cranial magnetic resonance imaging. CCT: cranial computed tomography. ^1^ Mean (min-max). ^2^ Median (min-max). ^3^ Numbers (%). ^4^ Pearson’s chi-squared test. ^5^ Mann-Whitney U test. ^6^ In patients without PET/CT, the time was calculated from the day of chest CT scan. ^7^ This information is missing in 2 patients.

Parameter	Pre-COVID Era (2012–2019), *n* = 77 Patients	COVID Era (2020–2021), *n* = 19 Patients	*p*-Value
Treated patients per month ^1^	0.9 (0–5)	1.5 (0–3)	0.04 ^5^
Multidisciplinary tumor board decision to SBRT [weeks] ^2,7^	4.0 (0–13.7)	6.4 (2.0–59.7)	0.005 ^5^
Bronchoscopy for staging ^3^	69 (89.6)	19 (100)	0.14 ^4^
Bronchoscopy to SBRT [weeks] ^2^	7.6 (3.3–23.1)	9.0 (5.9–62.7)	0.04 ^5^
Histological confirmation of diagnosis ^3^	63 (81.8)	15 (78.9)	0.77 ^4^
PET/CT for tumor Staging ^3^	68 (88.3)	15 (78.9)	0.29 ^4^
PET/CT or chest CT scan ^6^ to SBRT [weeks] ^2^	7.1 (2.3–49.1)	7.6 (4.3–60.0)	0.27 ^5^
PET/CT to SBRT [weeks] ^2^	7.0 (2.3–49.1)	7.4 (4.3–60.0)	0.76 ^5^
cMRI/CCT for staging ^3^	62 (80.5)	16 (84.2)	0.71 ^4^
Planning CT to SBRT [weeks] ^2^	1.7 (0.3–4.1)	3.0 (1.4–4.9)	<0.001 ^5^

**Table 3:** Cox regression analysis, influence of pretreatment diagnostic assessment on outcomes. The times for the parameters were calculated to the first day of stereotactic body radiation therapy (SBRT). The survival times were calculated from the first day of SBRT. HR: hazard ratio. OS: overall survival. PFS: progression-free survival. LRPFS: locoregional progression-free survival. LRC: locoregional control. DPFS: distant progression-free survival. DC: distant control. CI: confidence interval. PET/CT: positron emission tomography/computed tomography. CT: computed tomography. cMRI: cranial magnetic resonance imaging. CCT: cranial computed tomography. ^1^ This information is missing in 2 patients. ^2^ In patients without PET/CT, the time was calculated from the day of chest CT scan. ^3^ The cut-off (10 weeks) for the staging examinations was set in accordance with current studies on lung SBRT (e.g., PACIFIC-4 [22]).

	OS		PFS		LRPFS		LRC		DPFS		DC	
Parameter (Numbers of Patients)	HR (95% CI)	*p* Value	HR (95% CI)	*p* Value	HR (95% CI)	*p* Value	HR (95% CI)	*p* Value	HR (95% CI)	*p* Value	HR (95% CI)	*p* Value
Multidisciplinary tumor board decision to SBRT, weeks, median = 4.1, ≥median (*n* = 50) vs. <median (*n* = 44) ^1^	1.11 (0.63–1.98)	0.72	1.25 (0.72–2.19)	0.43	1.24 (0.71–2.19)	0.45	2.4 (0.6–9.65)	0.22	1.22 (0.69–2.14)	0.49	0.77 (0.22–2.64)	0.67
Bronchoscopy for staging, yes (*n* = 88) vs. no (*n* = 8)	0.96 (0.38–2.44)	0.93	1.08 (0.42–2.72)	0.88	1.05 (0.41–2.66)	0.92	23.36 (0–0.46 × 10^6^)	0.53	1.03 (0.41–2.62)	0.95	23.3 (0–0.19*10^6^)	0.49
Histological confirmation of diagnosis, yes (*n* = 78) vs. no (*n* = 18)	0.73 (0.37–1.45)	0.37	0.67 (0.35–1.28)	0.23	0.65 (0.34–1.24)	0.19	0.45 (0.11–1.79)	0.26	0.78 (0.4–1.53)	0.47	2.19 (0.28–17.12)	0.46
PET/CT for tumor staging, yes (*n* = 83) vs. no (*n* = 13)	0.39 (0.18–0.85)	0.02	0.34 (0.16–0.71)	<0.01	0.38 (0.18–0.82)	0.01	0.32 (0.07–1.58)	0.16	0.37 (0.18–0.77)	0.01	0.34 (0.07–1.63)	0.18
PET/CT or chest CT scan ^2^ to SBRT, weeks, ≥10 (*n* = 23) vs. <10 (*n* = 73) ^3^	1.41 (0.73–2.72)	0.31	1.59 (0.84–3.01)	0.15	1.69 (0.89–3.2)	0.11	5.26 (1.41–19.63)	0.01	1.27 (0.71–2.28)	0.42	0.81 (0.21–3.12)	0.76
PET/CT to SBRT, weeks, ≥10 (*n* = 17) vs. <10 (*n* = 66) ^3^	1.56 (0.76–3.2)	0.23	1.82 (0.91–3.64)	0.09	1.86 (0.93–3.72)	0.08	6.44 (1.44–28.78)	0.01	1.83 (0.91–3.68)	0.09	1.52 (0.31–7.33)	0.60
cMRI/CCT for staging, yes (*n* = 78) vs. no (*n* = 18)	0.8 (0.4–1.62)	0.54	0.77 (0.39–1.52)	0.46	0.75 (0.38–1.47)	0.40	0.84 (0.17–4.04)	0.83	0.71 (0.36–1.39)	0.32	0.53 (0.14–2)	0.35
Planning CT to SBRT, weeks, median = 1.86, ≥median (*n* = 53) vs. <median (*n* = 43)	0.62 (0.35–1.09)	0.10	0.72 (0.42–1.24)	0.23	0.71 (0.41–1.24)	0.23	1.58 (0.39–6.3)	0.52	0.67 (0.38–1.15)	0.15	0.43 (0.13–1.47)	0.18

## Data Availability

The data presented in this study are available on reasonable request from the corresponding author.

## Supplementary Materials

The following supporting information can be downloaded at: https://www.mdpi.com/article/10.3390/curroncol29020092/s1, Table S1: Comparison of dose fractionation schedules in the pre-COVID era and in the COVID era. Table S2: Comparison of pretreatment assessment between patients treated in the pre-COVID (Coronavirus Disease) era (here, defined from 2018–2019) and in the COVID era. Table S3: Cox regression analysis, influence of pretreatment diagnostic assessment on outcomes. Adjustment for multiple comparisons.
