# Supplementary material for: Patterns of Pretreatment Diagnostic Assessment in Patients Treated with Stereotactic Body Radiation Therapy (SBRT) for Non-Small Cell Lung Cancer (NSCLC): Special Characteristics in the COVID Pandemic and Influence on Outcomes
Source: Curr Oncol. 2022 Feb 13;29(2):1080–92. doi: 10.3390/curroncol29020092 (PMC8871078; doi:10.3390/curroncol29020092)
Supplement: Supplementary file 1 [file curroncol-29-00092-s001.zip › Table S2.pdf]

Table S2. Comparison of pretreatment assessment between patients treated in the pre-COVID (Coronavirus Disease) era (here, defined from 2018-2019) and in the COVID era. The times for the parameters were calculated to the first day of SBRT (stereotactic body radiation therapy). [1] Mean (min-max). [2] Median (min-max). [3] Mann-Whitney U test. [4] This information is missing in 2 patients.

**Parameter**	**Pre-COVID era (2018-2019), *n* = 26 patients**	**COVID era (2020-2021), *n* = 19 patients**	***p*-Value**
Treated patients per month [1]	1.1 (0–4)	1.5 (0–3)	0.25 [3]
Multidisciplinary tumor board decision to SBRT [weeks] [2,4]	5.0 (0–12.7)	6.4 (2.0–59.7)	0.26 [3]
Bronchoscopy to SBRT [weeks] [2]	8.0 (3.7–19.3)	9.0 (5.9–62.7)	0.16 [3]
Planning CT to SBRT [weeks] [2]	1.9 (1.0–3.0)	3.0 (1.4–4.9)	<0.001 [3]
